# Comparison of sGC activator and sGC stimulator in 5/6 nephrectomized rats on high-salt-diet

**DOI:** 10.3389/fphar.2024.1480186

**Published:** 2024-10-18

**Authors:** Xin Chen, Yingquan Xiong, Shufei Zeng, Denis Delić, Mohamed Gaballa, Philipp Kalk, Thomas Klein, Bernhard K. Krämer, Berthold Hocher

**Affiliations:** ^1^ Fifth Department of Medicine (Nephrology/Endocrinology/Rheumatology/Pneumology), University Medical Centre Mannheim, University of Heidelberg, Mannheim, Germany; ^2^ Department of Nephrology, Charité - Universitätsmedizin Berlin, Campus Mitte, Berlin, Germany; ^3^ Division of Nephrology, Nanfang Hospital, Southern Medical University, Guangzhou, China; ^4^ Translational Medicine and Clinical Pharmacology, Boehringer Ingelheim Pharma GmbH & Co. KG, Biberach, Germany; ^5^ Department of Pathology, Faculty of Veterinary Medicine, Benha University, Toukh, Egypt; ^6^ Academy of Scientific Research and Technology, Cairo, Egypt; ^7^ Department of Cardiometabolic Diseases Research, Boehringer Ingelheim Pharma GmbH & Co. KG, Biberach, Germany; ^8^ European Center for Angioscience, Medical Faculty Mannheim, University of Heidelberg, Mannheim, Germany; ^9^ Clinical Research Center for Reproduction and Genetics in Hunan Province, Reproductive and Genetic Hospital of CITIC-Xiangya, Changsha, Hunan, China; ^10^ Department of Endocrinology, IMD Institut für Medizinische Diagnostik Berlin-Potsdam GbR, Berlin, Germany; ^11^ Key Laboratory of Reproductive and Stem Cell Engineering, Central South University, Changsha, Hunan, China

**Keywords:** soluble guanylate cyclase activator, soluble guanylate cyclase stimulator, chronic kidney disease, renal fibrosis, apoptosis

## Abstract

**Introduction:**

Soluble guanylate cyclase (sGC) stimulators and activators are known to enhance kidney function in various models of chronic kidney disease (CKD) by increasing cyclic guanosine monophosphate (cGMP). Their differential effects on CKD progression, particularly under conditions of oxidative stress, remain unexplored by direct comparative studies.

**Methods:**

We conducted a side-by-side comparison using 5/6 nephrectomized rats on a high salt diet (5/6Nx+HSD) to evaluate the efficacy of the sGC stimulator BAY 41–8543 and the sGC activator BAY 60–2770 in CKD progression. BAY 41–8543 (1 mg/kg; twice daily) and BAY 60–2770 (1 mg/kg; once daily) were administered by gavage for 11 weeks.

**Results:**

The 5/6Nx+HSD model led to increased plasma creatinine, proteinuria, and blood pressure. Both BAY 41–8543 and BAY 60–2770 significantly reduced systolic and diastolic blood pressure to a similar extent but did not improve renal function parameters. Notably, BAY 60–2770 reduced renal fibrosis, including interstitial fibrosis and glomerulosclerosis, whereas BAY 41–8543 did not. These antifibrotic effects of BAY 60–2770 were independent of blood pressure reduction. Proteomic analysis revealed that BAY 60–2770 corrected the upregulation of 9 proteins associated with apoptosis and fibrosis, including Caspase-3, MKK6 (Mitogen-Activated Protein Kinase Kinase 6), Prdx5 (Peroxiredoxin-5), in the 5/6Nx+HSD group.

**Discussion:**

In contrast, BAY 41–8543 had no significant impact on these proteins. sGC activators were more effective than sGC stimulators in reducing renal fibrosis in 5/6 nephrectomized rats on a high salt diet, and this effect was due to modulation of apoptosis-associated proteins beyond the control of blood pressure.

## 1 Introduction

Chronic kidney disease (CKD) is a disease characterised by progressive impairment and loss of renal function (Webster et al., 2017). CKD involves complex pathological processes including oxidative stress, apoptosis, cell proliferation, inflammation, and fibrosis. After kidney injury, the important structural cells of the kidney (e.g., podocytes, tubular cells and endothelial cells) are lost due to apoptosis and necrosis, whereas the undesirable cells (e.g., myofibroblasts and inflammatory cells) proliferate in the injured tissue and lead to the extracellular matrix deposition (collagen and laminin), which is ultimately manifested as renal interstitial fibrosis, glomerulosclerosis, tubular atrophy and capillary thinning (Falke et al., 2015; Sanz et al., 2008). There are no therapeutic strategies that effectively degrade collagen to reverse renal fibrosis (Huang et al., 2023), so slowing renal fibrosis through pharmacological intervention early in the progression of CKD would be beneficial in preserving residual renal function.

cGMP (3′,5′-cyclic guanosine monophosphate), an important intracellular second messenger, has been shown to be strongly associated with renal fibrosis (Wang-Rosenke et al., 2008; Cui et al., 2014). The primary downstream effector of cGMP is PKG (cGMP dependent protein kinases), which exerts antifibrotic effects by inhibiting the profibrotic RhoA (Particular Rho protein)/ROCK (Rho associated protein kinase) and ERK1/2 (extracellular-signal regulated kinase) signaling pathways. This, in turn, suppresses TGF β (transforming growth factor β) signaling and prevents myofibroblast formation (Schinner et al., 2015; Shen et al., 2016). Many studies have reported a decrease in cGMP concentration after renal injury (Bai et al., 2007; Maimaitiyiming et al., 2013), and an increase in cGMP concentration is beneficial in improving renal injury (Krishnan et al., 2018; Stehle et al., 2022). Physiologically, NO (nitric oxide) - sGC (soluble guanylate cyclase) – cGMP is an important pathway for renal cGMP production. NO stimulates sGC by binding to sGC, which catalyzes the conversion of guanosine triphosphate (GTP) to cGMP, thereby initiating a series of biological responses, including antifibrotic (Schinner et al., 2015), antiproliferative and anti-inflammatory effects (Krishnan et al., 2018; Harloff et al., 2022). sGC is the key enzyme in this pathway and consists of an α-subunit, a β-subunit, with a heme group as binding motif for NO (Nossaman et al., 2012). The heme structural domain of sGC is key to its regulation, and this structural domain contains not only NO stimulation sites, but also sites for NO-independent sGC activators. Under conditions of renal injury (oxidative stress), oxidative stress leads to a change in the redox state of the heme structural domain of the sGC (from ferrous to ferric), rendering the sGC unresponsive to NO (Stasch et al., 2015). Further, NO is lost due to oxidation by oxygen radicals. Impaired formation and bioavailability of NO may be responsible for cGMP deficiency in pathological conditions. Two classes of compounds have been developed to increase cGMP production - sGC stimulators and sGC activators. sGC stimulators directly stimulate the non-NO-dependent sites of reduced-state sGCs and synergise with NO by stabilising reduced-type sGCs. In contrast, sGC activators directly activate oxidised/heme-free sGCs and have a superimposed effect with NO(15). Both classes of compounds have now demonstrated nephroprotective effects in many studies, including improvements in proteinuria and renal fibrosis (Wang-Rosenke et al., 2011; Kalk et al., 2006; Czirok et al., 2017). In some models of disease associated with oxidative stress, oxidised or haemoglobin-free sGC levels are elevated (Stasch et al., 2011). Thus, sGC activators may be more advantageous in CKD. To verify this inference, we applied sGC stimulator (BAY 41–8543) and sGC activator (BAY 60–2770) in 5/6 nephrectomized + high salt diet rats and explored their effectiveness and differences histologically and proteomically.

## 2 Materials and methods

### 2.1 Experimental animals and protocol

The animal experiments were approved by the Animal Care and Use Committee of Jinan University, Guangzhou, China (IACUC-20170904092822). All animal experiments complied with the ARRIVE guidelines and were carried out in accordance with the U.K. Animals (Scientific Procedures) Act, 1986 and associated guidelines, as well as the National Research Council’s Guide for the Care and Use of Laboratory Animals.

**FIGURE 1:**
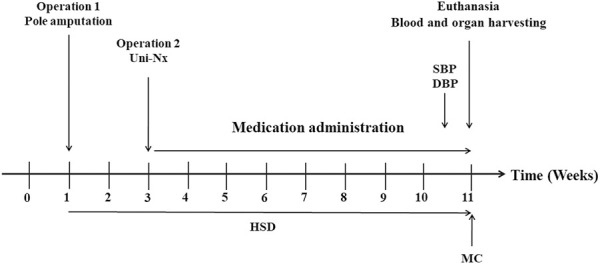
Time course of the animal study. SBP: Systolic Blood Pressure; DBP: Diastolic Blood Pressure; HSD: High Salt Diet; MC: Metabolic Cages; Poles amputation: Amputation of both poles of left kidney; Uni-Nx: Unilateral nephrectomy of the right kidney.

75 6-week-old male Wistar rats were purchased from animal experiment center of Guangzhou University of Chinese Medicine. After a 10-day acclimatisation the animals were assigned to the following groups: Sham + ND (Normal Diet)+ PBO (Placebo) (n = 15); 5/6Nx (5/6 nephrectomy) + HSD (High Salt Diet) + PBO (n = 30); 5/6Nx + HSD + BAY 41–8543 (1 mg/kg; twice per day) (n = 15); 5/6Nx + HSD + BAY 60–2770 (1 mg/kg; once per day) (n = 15). The normal diet was according to the AIN93M criteria (Reeves et al., 1993). High-salt diets were adjusted to 2% sodium chloride on the AIN93M standard. 5/6Nx was performed on rats under anesthesia with pentobarbital sodium (36–39 mg/kg; intraperitoneally) as follows: amputation of the poles of the right kidney in week 1 followed by uni-nephrectomy of the left kidney (Uni-Nx) in week 3 (Figure 1). After surgery, penicillin G 20 000U was given intramuscularly to prevent infection. At the same time points, all other operations except nephrectomy were performed on rats in the Sham + ND + PBO group. All groups except Sham + ND + PBO were placed on a high-salt diet from operation 1 until euthanasia (Figure 1). All rats received medication/placebo by gavage from the time of surgery until before euthanasia. The sGC sitmulator BAY 41–8543 and the sGC activator BAY 60–2770 were manufactured by Bayer, Pharmaceuticals AG (Wuppertal, Germany), exhibit the typical profile of an sGC stimulator and sGC activator but are non-clinical tool compounds and could not be used in humans. The blood pressure (BP) and heart rate of the animals were assessed at 11 weeks using non-invasive tail cuff plethysmography of the tail artery (IITC Life Science). Rats were habituated to the BP measurement device for 7 days. At week 11 of the study, the animals were placed in metabolic cages to collect 24-h urine. All rats were euthanised at week 11, after which blood and organ (kidney) samples were collected.

### 2.2 Plasma and urine parameters measurement

Levels of plasma creatinine, glucose, and insulin as well as urinary creatinine, and urinary protein were detected by an automated clinical chemistry analyzing system (Roche cobas 6800, Roche Ltd., Switzerland). Urinary albumin (ab235642, Abcam, Cambridge, United Kingdom) and plasma B-type natriuretic peptide (BNP45) (ab108816, Abcam, Cambridge, United Kingdom) levels were determined quantitatively using Enzyme-Linked Immunosorbent Assay kit.

### 2.3 Histological analysis

Paraffin-embedded kidney tissue was cut into 2 µm slices and stained with Sirius red and Periodic Acid–Schiff (PAS). Immunofluorescence was used to assess the expression of renal type I collagen (Catalogue No. 2150–1908, Bio-Rad Laboratories). Approximately 80% of the area of each immunofluorescent section was acquired and analysed using a fluorescence microscope with a magnification of ×200. The relative fluorescence units of type I collagen were assessed by ImageJ software (National Institutes of Health, United States). The glomerulosclerosis index was assessed by two investigators using a subjective, semi-quantitative scoring system (grades I-IV) to evaluate the percentage of periodic acid schiff-positive areas within the glomerulus of PAS-stained slices. The mean of the two scoring results was used. On each PAS-stained slice, 40–75 glomeruli were analysed to assess the glomerular size by using ImageJ software. The percentage of renal interstitial fibrosis was analysed using Sirius Red-stained images and using the ImageJ threshold method (National Institutes of Health, United States), as previously described (Kanasaki et al., 2014). All analyses covered more than 80% of the area of each slice.

### 2.4 Liquid chromatography and mass spectrometry (proteomics)

Proteins were extracted from plasma, with each group comprising 10–19 samples. This extraction was conducted through reversed-phase liquid chromatography, followed by matrix-assisted laser desorption ionization mass spectrometry analysis. Subsequently, acquired mass spectrometric data were analyzed to identify signals that were differentially regulated among the groups. Amino acid sequences corresponding to these differentially regulated signals were identified using methods previously described (Tsuprykov et al., 2016).

### 2.5 Statistical analysis

Normally distributed data were given as mean ± SEM. Non-normally distributed data were presented as median (25th–75th percentile). Analyses were performed in Prism 8.3 (GraphPad, La Jolla, CA, United States). For between-group comparisons, we used one-way ANOVA with Bonferroni’s *post hoc* test to analyse normally distributed data, and Kruskal-Wallis test with Dunn’s *post hoc* test for non-normally distributed data. Differences were considered as statistically significant if p< 0.05.

## 3 Results

### 3.1 BAY 41–8543 and BAY 60–2770 did not improve renal function in 5/6 nephrectomized rats on a high-salt-diet

5/6Nx+HSD+PBO caused a decrease in Ccr (creatinine clearance), an increase in plasma creatinine, proteinuria, blood pressure, relative heart weight and relative kidney weight, and no effect on plasma insulin, plasma glucose, plasma BNP45 or body weight in rats (Table 1). BAY 41–8543 and BAY 60–2770 treatment had no effect on these renal function and glucose-related parameters, but both significantly reduced SBP (systolic blood pressure) and DBP (diastolic blood pressure) (Table 1). Rats in the BAY 41–8543 and BAY 60–2770 treatment groups both gained weight more slowly than those in the 5/6Nx+HSD+PBO group (Table 1). The 5/6Nx+HSD model did not alter plasma insulin or glucose levels in rats. Similarly, treatment with BAY 41–8543 or BAY 60–2770 did not affect plasma insulin levels in non-diabetic nephropathy rats. While BAY 60–2770 had no effect on plasma glucose, a slight decrease in glucose levels was observed in the BAY 41–8543 treatment group compared to the 5/6Nx+HSD+PBO group. 5/6Nx + HSD led to increased 24-h urinary protein excretion and ACR; however, neither drug intervention improved proteinuria. The ACR data showed significant within-group variability and low statistical power.

**TABLE 1:** Basic animal characteristics.

Parameters	Sham + ND + PBO (n = 12–13)	5/6Nx + HSD + PBO (n = 15–23)	5/6Nx + HSD + BAY 41–8543 (n = 11–15)	5/6Nx + HSD + BAY 60–2770 (n = 13–15)
Body weight(g)	472.10 (445.85–503.75)	440.10 (417.20–474.40)	380.80 (359.85–411.25)**	379.70 (355.68–436.78)*
Rel. heart weight (mg/g)	2.72 (2.55–2.92)***	3.30 (3.08–4.23)	4.01 (3.33–4.39)	4.25 (3.15–5.20)
Rel. kidney weight (mg/g)	3.23 ± 0.08*	3.96 ± 0.23	3.74 ± 0.15	3.87 ± 0.22
SBP (mmHg)	124.66 (118.33–130.50)***	153.00 (149.00–163.66)	123.67 (120.08–133.50)***	130.50 (127.17–133.00)***
DBP (mmHg)	101.60 ± 2.43****	123.60 ± 2.21	99.77 ± 3.59****	91.08 ± 2.15****
Plasma creatinine (umol/L)	47.00 (45.00–49.00)***	75.00 (69.00–86.00)	87.00 (80.50–112.50)	88.00 (82.50–102.30)
24 h urinary protein excretion (mg/24 h)	4.81 (4.23–5.79)****	11.63 (7.48–36.08)	13.94 (7.42–27.12)	8.82 (4.93–39.10)
ACR (mg/mmoL)	1.51 (1.27–2.27)****	38.04 (11.03–199.43)	80.18 (26.21–270.56)	43.50 (14.80–64.31)
Ccr (mL/min)	1.06 (0.62–1.48)*	0.82 (0.66–0.99)	0.56 (0.46–0.67)	0.56 (0.37–0.67)
Plasma insulin (ug/L)	5.86 (1.31–6.54)	4.55 (1.10–12.87)	2.72 (0.74–3.55)	7.85 (2.59–19.43)
Plasma glucose (mmol/L)	6.08 (5.62–7.18)	6.52 (5.36–7.69)	5.29 (4.98–5.91)*	7.41 (6.67–7.81)
Plasma BNP45 (ng/mL)	2.26 ± 0.49	2.13 ± 0.32	2.14 ± 0.25	1.96 ± 0.15

Note: Rel. heart weight (Relative heart weight) = heart weight/final body weight; Rel. kidney weight (Relative kidney weight) = kidney weight/final body weight; SBP: systolic blood pressure; DBP: diastolic blood pressure; ACR (urinary albumin to creatinine ratio, mg/mmol) = urinary albuminuria/urinary creatinine; Ccr (creatinine clearance) = [urinary creatinine (μmol/L) * urinary flow (mL/min)]/serum creatinine (μmol/L); BNP45: B-type natriuretic peptide; Sham: Sham operation; 5/6Nx: 5/6 nephrectomy model; PBO: placebo; ND: normal diet; HSD: High Salt Diet. Normally distributed data were given as mean ± SEM., Non-normally distributed data were presented as median (25th–75th percentile). *p< 0.05; **p< 0.01; ***p< 0.001; ****p< 0.0001, significantly different from 5/6Nx + HSD + PBO.

### 3.2 BAY 60–2770 more significantly improves renal fibrosis than BAY 41–8543 in 5/6 nephrectomized rats on a high-salt-diet

**FIGURE 2:**
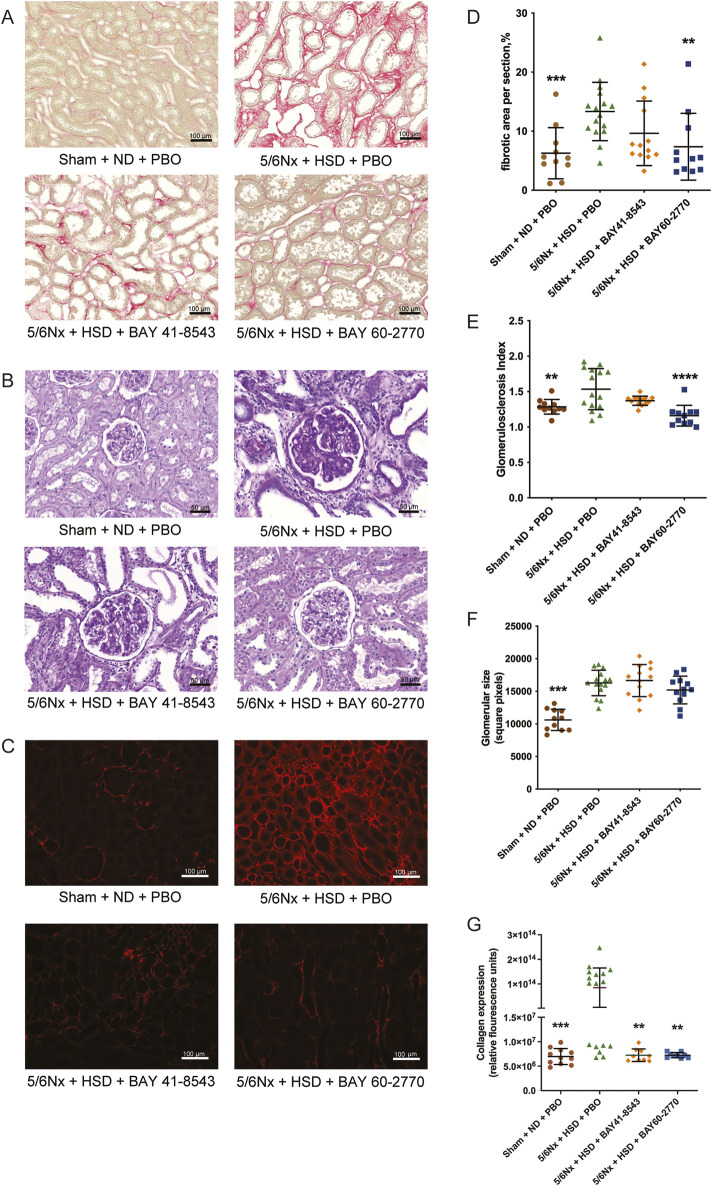
Kidney morphology. **(A)** Photomicrographs of sirius red-stained kidneys for the detection of fibrosis, scale bar = 100 μm. **(B)** Photomicrographs of periodic acid Schiff-stained kidneys for the detection of glomerulosclerosis and glomerular size, scale bar = 50 μm. **(C)** Photomicrographs of collagen I immunostaining, the red color indicates the area of collagen deposition, scale bar = 100 μm. **(D)** Renal interstitial fibrosis. **(E)** Glomerulosclerosis index. **(F)** Glomerular size. **(G)** Immunofluorescence analysis of renal collagen I. Sham: Sham operation; 5/6Nx: 5/6 nephrectomy model; PBO: Placebo; ND: Normal Diet; HSD: High Salt Diet. *p< 0.05; **p< 0.01; ***p< 0.001; ****p< 0.0001, significantly different from 5/6Nx + HSD + PBO.

Histological analysis (Figures 2A–C) showed that 5/6 nephrectomy with high salt diet resulted in severe interstitial fibrosis (Figures 2A, D), glomerulosclerosis (Figures 2B, E), enlarged glomeruli (Figures 2B, F) and type I collagen deposition (Figures 2C, G). Immunofluorescence analysis showed that both BAY 41–8543 and BAY 60–2770 decreased type I collagen expression (Figures 2C, G). However, only BAY 60–2770 ameliorated renal interstitial fibrosis (Figures 2A, D) and glomerulosclerosis (Figures 2B, E).

### 3.3 BAY 60–2770 more significantly downregulates the expression of apoptosis-related proteins than BAY 41–8543 in 5/6 nephrectomized rats on a high-salt-diet

92 proteins were detected in rat blood. At the screening criterion of p< 0.05, 5/6Nx + HSD resulted in the aberrant expression of 56 proteins, whereas at the screening criterion of p< 0.01, 5/6Nx + HSD resulted in the aberrant expression of 19 proteins. The proteins that were simultaneously regulated in the model group (5/6Nx + HSD + PBO) versus the treatment group (5/6Nx + HSD + BAY 41–8543 and 5/6Nx + HSD + BAY 60–2770) according to the p< 0.01 screening criteria are presented in Table 2. BAY 60–2770 ameliorated 9 proteins that were aberrantly expressed due to 5/6Nx + HSD, including Caspase-3, MKK6 (Mitogen-Activated Protein Kinase Kinase 6), Prdx5 (Peroxiredoxin-5), Axin1 (Axis Inhibition Protein 1), Follistatin, Wfikkn2 (WAP, Follistatin/Kazal, Immunoglobulin, Kunitz And Netrin Domain Containing 2), Qdpr (Quinoid Dihydropteridine Reductase), Ddah1 (Dimethylarginine Dimethylaminohydrolase 1), and Apbb1ip (Amyloid Beta Precursor Protein Binding Family B Member 1 Interacting Protein). Of these 9 proteins, 7 were found to be associated with apoptosis and fibrosis, including Caspase-3, MKK6, Prdx5, Axin1, Follistatin, Ddah1 and Apbb1ip. However, there is no observed effect of BAY 41–8543.

**TABLE 2:** Results of proteomic analyses in plasma.

Peptides	Sham + ND + PBO (n = 10–13)	5/6Nx + HSD + PBO (n = 13–19)	5/6Nx + HSD + BAY 41–8543 (n = 10–14)	5/6Nx + HSD + BAY 60–2770 (n = 10)
Caspase-3	9.31 (8.25–10.00)**	10.05 (9.75–10.44)	9.80 (9.50–10.07)	8.90 (8.32–9.64)***
MKK6	5.60 ± 0.25**	6.52 ± 0.11	6.28 ± 0.14	5.23 ± 0.38**
Prdx5	6.42 (5.49–7.35)***	7.52 (7.23–8.24)	7.18 (6.85–7.67)	6.58 (5.63–7.16)***
Axin1	4.72 ± 0.26**	5.69 ± 0.14-	5.38 ± 0.14-	4.51 ± 0.28***
Follistatin	8.43 ± 0.27**	9.53 ± 0.22	9.18 ± 0.19	8.33 ± 0.11**
Wfikkn2	7.74 ± 0.05****	8.08 ± 0.04	8.05 ± 0.05	7.87 ± 0.04**
Qdpr	9.31 ± 0.13***	10.00 ± 0.10	9.86 ± 0.08	9.21 ± 0.24***
Ddah1	1.68 ± 0.15***	2.54 ± 0.14	2.35 ± 0.11	1.68 ± 0.18***
Apbb1ip	0.93 ± 0.14***	1.71 ± 0.14	1.33 ± 0.11	0.93 ± 0.15***

Note: MKK6 (Mitogen-Activated Protein Kinase Kinase 6), Prdx5 (Peroxiredoxin-5), Axin1 (Axis Inhibition Protein 1), Wfikkn2 (WAP, Follistatin/Kazal, Immunoglobulin, Kunitz And Netrin Domain Containing 2), Qdpr (Quinoid Dihydropteridine Reductase), Ddah1 (Dimethylarginine Dimethylaminohydrolase 1) Apbb1ip (Amyloid Beta Precursor Protein Binding Family B Member 1 Interacting Protein). Sham: Sham operation; 5/6Nx: 5/6 nephrectomy model; PBO: placebo; ND: normal diet; HSD: High Salt Diet. Normally distributed data were given as mean ± SEM., Non-normally distributed data were presented as median (25th–75th percentile). *p< 0.05; **p< 0.01; ***p< 0.001; ****p< 0.0001, significantly different from 5/6Nx + HSD + PBO.

## 4 Discussion

We explored for the first time the renoprotective mechanisms of sGC stimulator and activator in a 5/6 nephrectomy high-salt diet model in a side-by-side comparison. We have chosen low dosage of both drugs with moderate but comparable blood pressure effects in an aggressive CKD model characterized by pronounced kidney fibrosis to explore potential underlying differences in the molecular mechanisms of sGC activators versus stimulator using a non-hypothesis driven approach: proteomics. Both sGC stimulator and activator reduced the elevated blood pressure in rats caused by a 5/6 nephrectomy on high-salt diet to almost the same extent. Although the sGC stimulator and sGC activator could not improve kidney function (assessed by proteinuria), the sGC activator is more effective than sGC stimulator against renal fibrosis, including attenuating renal interstitial fibrosis and glomerulosclerosis. Proteomic analysis suggests that the anti-fibrotic effects of the sGC activator are linked to effects of apoptosis regulating proteins such as Caspase-3, MKK6, Prdx5, Axin1, Follistatin, Ddah1, and Apbb1ip in addition to beneficial effects on blood pressure control.

sGC stimulator and activator have been shown in many current studies to have nephroprotective effects (Wang-Rosenke et al., 2011; Kalk et al., 2006; Czirok et al., 2017). However, no improvement in renal function indices (e.g., plasma creatinine and proteinuria) was observed in our study. The therapeutic dose of the medication and the severity of the disease model may be important factors to explain this. In the currently published preclinical studies supporting the nephroprotective effects of sGC stimulators and activators, most of the oral therapeutic doses of BAY 41–8543 were 10 mg/kg/d (Wang-Rosenke et al., 2011; Wang-Rosenke et al., 2012; Stancu et al., 2015), whereas the oral doses of BAY 60–2770 ranged from 0.3 to 3 mg/kg/d (Sharma et al., 2023; Geenen et al., 2016). Furthermore, the effects of both BAY 41–8543 and BAY 60–2770 were revealed to be dose-dependent (Sharma et al., 2023; Stasch et al., 2002; Frees et al., 2021). In this study, we explored whether BAY 41–8543 and BAY 60–2770 could exert a nephroprotective effect at lower doses while avoiding excessive effects on blood pressure, so we chose lower doses of BAY 41–8543 (1 mg/kg; twice per day) and the sGC activator BAY 60–2770 (1 mg/kg; once per day). The study applied an extremely severe model of renal injury - 5/6 nephrectomy with a high salt diet - which may also have limited the observation of therapeutic effects of the low doses. In addition, improvement in functional parameters may also lag behind histological parameters. Immunofluorescence analysis showed that both BAY 41–8543 and BAY 60–2770 reduced renal type I collagen deposition. Interestingly, separate assessment of interstitial fibrosis and glomerulosclerosis showed significant improvement with the sGC activator BAY 60–2770 but not with the sGC stimulator BAY 41–8543. This is consistent with another study demonstrating that sGC activators alleviate interstitial fibrosis and glomerulosclerosis (Reinhart et al., 2023). Extracellular matrix deposition mainly contains type IV collagen, type I collagen, and laminin (Bulow and Boor, 2019), and therefore we consider that the expression of type I collagen does not fully represent the severity of renal fibrosis. Overall, sGC activators were superior to sGC stimulators in a more severe model of kidney injury (5/6 nephrectomy with high salt diet). A preclinical study in a side-by-side comparative study also demonstrated that sGC activators were more effective than sGC stimulators in the treatment of diabetes-related vascular and renal complications (Sharma et al., 2023). Another side-by-side study also suggested that sGC activators were superior to sGC stimulators in improving function and structure in rats with diabetic nephropathy (Balzer et al., 2023). Their nephroprotective effects are also thought to be related to their vasodilatory effects by increasing cGMP. In our study, BAY 41–8543 and BAY 60–2770 also attenuated blood pressure in 5/6 nephrectomised rats on a high-salt diet to almost the same extent, but the renal prognosis was inconsistent. The superiority of BAY 60–2770 in antifibrotic may be mediated by the modulation of apoptosis.

Apoptosis is an essential part of the fibrous process in the kidney. Activation of apoptosis in the kidney results in the loss of kidney resident cells (e.g., podocytes, tubular cells, and endothelial cells), as well as the proliferation and/or recruitment of undesirable cells (e.g., myofibroblasts and inflammatory cells). Normal tissue architecture is replaced by fibroblasts as well as deposited extracellular matrix, which ultimately manifests as renal interstitial fibrosis, glomerulosclerosis, tubular atrophy and capillary thinning (Sanz et al., 2008; Savill, 1994). Caspase-3 is a key executor of apoptosis (Sanz et al., 2023). Various pro-apoptotic factors activate downstream Caspase-3 cleavage of gasdermin E (GSDME), leading to extensive protein hydrolysis and resulting in cell death. It has been demonstrated that caspase-3−/− mice have reduced renal fibrosis, decreased α-smooth muscle actin expression and reduced collagen deposition in peritubular capillaries at 3 weeks after ischemia-reperfusion injury (Yang et al., 2018). In addition, MKK6, a mitogen-activated protein kinases (MAPKs) kinase, activates p38 MAPK in response to inflammatory cytokines or environmental stresses to induce apoptosis and fibrosis (Matsuzawa et al., 2002; Zhang et al., 2020). Some current studies show that MMK6/p38 MAPK-induced apoptosis is mediated by activation of the downstream effector caspase-3 (Jeong et al., 2014; Machino et al., 2003; Noguchi et al., 2000; Iyoda et al., 2003). In our present study, proteomic analyses showed that circulating MMK6 and caspase-3 were increased after a 5/6 nephrectomy high-salt diet and significantly decreased after intervention with sGC activator BAY 60–2770, while renal interstitial fibrosis and glomerulosclerosis were also reduced in this treatment group. In addition to MMK6 and Caspase-3 proteins, 5 of the 9 proteins regulated by BAY 60–2770 are involved in the regulation of apoptosis, including Prdx5, Axin1, Follistatin, Ddah1, and Apbb1ip. These proteins are not key proteins in the regulation of apoptosis, and the specific mechanism of their involvement in apoptosis remains unclear. Overexpression of PRDX5 was reported to inhibit apoptosis by scavenging ROS and reactive nitrogen (Xie et al., 2022; De Simoni et al., 2013). Follistatin was also reported to prevent apoptosis (Mehta et al., 2019; Hansen et al., 2016). Axin1 is thought to promote apoptosis in hepatocellular carcinoma cells and cardiomyocytes (Li et al., 2013; Ye et al., 2017). The relationship between DDAH1 and apoptosis is controversial. It has been suggested that DDAH1-mediated nitric oxide production in human lung microvascular endothelial cells promotes apoptosis in lung smooth muscle cells (Almazroue et al., 2023), whereas some studies have suggested that the lack of DDAH1 enhances cellular sensitivity to pro-apoptotic factors (Zhao et al., 2016; Lumicisi et al., 2015). It has been suggested that knockdown of circular APBB1IP upregulates caspase-3 protein expression and increases apoptosis (Mo et al., 2020). In summary, sGC activator BAY 60–2770 regulate apoptosis through the above seven proteins in an inconsistent direction, but Caspase-3, a key executor of apoptosis, was significantly downregulated. In particular, sGC has been shown to be expressed in the glomerular mesangium and glomerular arterioles and even in mesangial fibroblasts (Theilig et al., 2001). Therefore, we concluded that sGC activator inhibited apoptosis in general, which in turn attenuated renal fibrosis. Activation of the NO/cGMP pathway has also now been proposed to favour inhibition of apoptosis (Ha et al., 2003; Lee et al., 2019). The mechanism by which sGC activator inhibits apoptosis is likely related to its increased production of cGMP and inhibition of caspase-3 and MKK6 expression. However, sGC stimulator BAY 41–8543 intervention had no effect on the expression of these proteins, including MMK6 and caspase-3, nor on renal interstitial fibrosis and glomerulosclerosis. We suggest that sGC activators are more effective than sGC stimulators in protecting the kidney in a model of severe kidney injury, and that the advantage of sGC activators is likely to be related to the regulation of apoptosis. In fact, two recent clinical trials (NCT04750577 and NCT04736628) on the novel, potent sGC activator avenciguat have demonstrated that it improves albuminuria in patients with CKD, both with and without type 2 diabetes mellitus (Heerspink et al., 2024).

## 5 Limitations

We conducted side-by-side comparative studies for sGC stimulators (BAY 41–8543) and activators (BAY 60–2770), but we did not perform dose-response relationships and only designed treatment groups with a low dose and therefore could not determine whether the results were affected by the chosen doses. Assessing blood and renal cGMP levels would provide valuable insight into whether the effects of sGC activators on apoptosis are linked to their primary pharmacological mechanism (NO/sGC/cGMP). The 5/6 neprectomy model with a substantial inflammatory response might cause an unusual high oxidative stress burden, rendering the sGC activators more efficacious. Therefore, oxidative stress markers (e.g., 8-isoprostane F2α, superoxide dismutase, catalase and glutathione peroxidase) could help to determine whether oxidative stress is a major factor contributing to the stronger effects of sGC activators over sGC stimulators. The results of type I collagen in this study did not fully correlate with the results of the scores for renal interstitial fibrosis and glomerulosclerosis, and it is recommended that future studies do not overlook the importance of IV collagen when assessing the extent of renal fibrosis. Proteomic analysis was performed only on rat blood, which provides valuable insights within the specific 5/6 nephrectomy high-salt diet model, but is less conclusive compared to proteomic data from kidney tissue.

## 6 Conclusion

This study provides a direct comparison of the effects of the sGC stimulator BAY 41–8543 and the sGC activator BAY 60–2770 in a severe CKD model induced by 5/6 nephrectomy and a high salt diet. Both compounds effectively reduced systolic and diastolic blood pressure to a similar extent, yet only BAY 60–2770 demonstrated significant antifibrotic properties, including the reduction of interstitial fibrosis and glomerulosclerosis. These effects were independent of the blood pressure-lowering action, highlighting a distinctive mechanism of BAY 60–2770 involving the modulation of apoptosis-related proteins such as Caspase-3 and MKK6. In contrast, BAY 41–8543 showed no significant impact on these proteins or renal fibrosis.

The fundamental molecular difference between sGC activators and stimulators lies in their modes of action. sGC stimulators, like BAY 41–8543, directly stimulate the reduced state of sGC, synergizing with NO to stabilize the enzyme and enhance cGMP production. On the other hand, sGC activators, such as BAY 60–2770, directly activate the oxidized or heme-free state of sGC, independent of NO, making them particularly effective under conditions of oxidative stress where NO availability is compromised.

One of the key strengths of this study is the use of a robust and severe CKD model, which closely mimics the high oxidative stress conditions seen in human CKD. Additionally, the side-by-side comparison under controlled experimental conditions provides clear insights into the differential effects of sGC stimulators and activators. The comprehensive proteomic analysis further elucidates the molecular mechanisms underlying the observed effects.

Thus, our findings suggest that sGC activators, particularly BAY 60–2770, may offer superior therapeutic benefits in CKD, especially in conditions marked by high oxidative stress. These results emphasize the potential for sGC activators to provide targeted antifibrotic therapy beyond blood pressure control, paving the way for improved treatment strategies in managing CKD progression.

## Data Availability

The datasets presented in this study can be found in online repositories. The names of the repository/repositories and accession number(s) can be found in the article/supplementary material.
